# Ferric Sulfate and Proline Enhance Heavy-Metal Tolerance of Halophilic/Halotolerant Soil Microorganisms and Their Bioremediation Potential for Spilled-Oil Under Multiple Stresses

**DOI:** 10.3389/fmicb.2018.00394

**Published:** 2018-03-07

**Authors:** Dina M. Al-Mailem, Mohamed Eliyas, Samir S. Radwan

**Affiliations:** Microbiology Program, Department of Biological Sciences, Faculty of Science, Kuwait University, Safat, Kuwait

**Keywords:** bioremediation, haloarchaea, halophilic bacteria, heavy metals, hypersaline, environments, oil bioremediation

## Abstract

The aim of this study was to explore the heavy-metal resistance and hydrocarbonoclastic potential of microorganisms in a hypersaline soil. For this, hydrocarbonoclastic microorganisms were counted on a mineral medium with oil vapor as a sole carbon source in the presence of increasing concentrations of ZnSO_4_, HgCl_2_, CdSO_4_, PbNO_3_, CuSO_4_, and Na_2_HAsO_4._ The colony-forming units counted decreased in number from about 150 g^-1^ on the heavy-metal-free medium to zero units on media with 40–100 mg l^-1^ of HgCl_2_, CdSO_4_, PbNO_3_, or Na_2_HAsO_4_. On media with CuSO_4_ or ZnSO_4_ on the other hand, numbers increased first reaching maxima on media with 50 mg l^-1^ CuSO_4_ and 90 mg l^-1^ ZnSO_4_. Higher concentrations reduced the numbers, which however, still remained considerable. Pure microbial isolates in cultures tolerated 200–1600 mg l^-1^ of HgCl_2_, CdSO_4_, PbNO_3_, CuSO_4_, and Na_2_HAsO_4_ in the absence of crude oil. In the presence of oil vapor, the isolates tolerated much lower concentrations of the heavy metals, only 10–80 mg l^-1^. The addition of 10 Fe_2_(SO_4_)_3_ and 200 mg l^-1^ proline (by up to two- to threefold) enhanced the tolerance of several isolates to heavy metals, and consequently their potential for oil biodegradation in their presence. The results are useful in designing bioremediation technologies for oil spilled in hypersaline areas.

## Introduction

Oil started to be extensively produced and used as energy and raw-material sources since about 80 years. This was associated with increasing rates of pollution of terrestrial, aquatic, and atmospheric environments with spilled oil, oil vapor, and processed oil products. It has been estimated that alone the marine environment is charged yearly with 10 Mt of hydrocarbon pollutants (Banerjee et al., 2006). As expected, pollution rates are particularly high in oil-producing countries such as the Arabian Gulf states. The Gulf water-body is used by vessels that carry about 50% of the marine-transported oil worldwide (Hunter, 1982). Those states belong geographically to the semiarid region and are characterized by harsh climates. For example, the temperature in the small state of Kuwait during the long, dry summer frequently exceeds 50°C. Under such harsh conditions, oil bioremediation and self-cleaning via activities of nonextremophilic microorganisms are minimal (Margesin and Schinner, 2001; Oren, 2002). At such high temperatures, coastal seawaters trapped during tidal movement lose water by excessive evaporation, and the respective areas become thus hypersaline with NaCl concentrations reaching 4 M and higher. Like elsewhere (Lefebvre and Moletta, 2006), the hypersaline coastal areas in Kuwait are subjected to contamination with spilled oil. Bioremediation of such polluted areas cannot be achieved by nonextremophilic microorganisms (Pieper and Reineke, 2000; Oren, 2002); only halophilic/halotolerant microorganisms could be effective.

Halophilic/halotolerant microorganisms with hydrocarbon-utilization potential were recorded and isolated from hypersaline areas all over the globe (Oren et al., 1992; Emerson et al., 1994; Margesin and Schinner, 2001; Garcia et al., 2004; Nicholson and Fathepure, 2005; Zhao et al., 2009; Bonfa et al., 2011; Fathepure, 2014). Those hydrocarbonoclastic halophiles include prokaryotes (bacteria and archaea) and eukaryotes (yeasts and molds). Our group in Kuwait contributed to such studies and published extensive information on numbers, identities, and hydrocarbonoclastic potential of hydrocarbonoclastic microorganisms from hypersaline coastal areas in this region (Al-Mailem et al., 2012, 2014a). Emphasis was put on the feasibility of using these indigenous microorganisms in bioremediation and self-cleaning of local oil spills. The Gulf microorganisms naturally are stressed, not only by high salinity, harsh climatic conditions, and oil spills, but also by toxic heavy metals that may be associated with crude oil (Osuji and Onojake, 2004; Basumatary et al., 2012). Within this context, toxicity and resistance of heavy metals have been studied using predominantly nonextremophilic microorganisms (for review, see Nies, 1999). Those metals have a density above 5 g cm^-3^ (atomic weights > 50 amu) (Weast, 1984). They potentially form unspecific complex compounds in the cell which leads to toxicity. Yet, some heavy metals are essential trace elements. Two systems for heavy-metal uptake are known. The fast, unspecific system which is constitutively expressed; it depends solely on concentration gradient force. The second is the slow uptake system, with high substrate specificity in which ATP is consumed. Heavy-metal toxicity arises when the heavy-metal cations bind to SH groups leading to the inhibition of the activity of sensitive (especially respiratory) enzymes. Ions may also bind with O_2_ leading to oxidative stress. Three mechanisms for heavy-metal resistance are known. The first involves the active extrusion (efflux) of ions from the cell. The second is the segregation of cations (especially the S-lovers) into complex compounds by thiol-containing molecules. Thirdly, metal ions may be reduced to a less toxic oxidation (sometimes volatile, e.g., Hg^o^) state.

Within a research project on the bioremediation of oily hypersaline regions in Kuwait, we reported recently on biostimulation of pure, hydrocarbonoclastic microorganisms from those habitats by the addition of cations including Fe^3+^ (Al-Mailem et al., 2017). The major objective of this current study was to elaborate on the previous work by investigating the sensitivity and tolerance of the same microorganisms to common heavy metals, and to measure the effects of individual heavy metals on crude-oil removal by such microorganisms under salt stress. In other words, the main aim was jointly exploring the heavy-metal tolerance and hydrocarbon-degrading potential of novel halotolerant/halophilic isolates from hypersaline coastal soil in Kuwait. We started this study by counting the numbers of colony-forming units (CFU) of hydrocarbonoclastic, halophilic/halotolerant microorganisms for the hypersaline soil on media containing increasing concentrations of heavy metals. This part was followed by another one in which pure cultures from the hypersaline soil were used. It was also proposed to investigate whether or not, Fe^3+^ and proline (a compatible solute) may affect the microbial tolerance to the toxicity of individual heavy metals. Information gained from this research would help in designing biotechnologies for bioremediation of spilled oil in areas under multiple stresses. Within this context, our group recently found that calcium and dipicolinic acid (constituents of heat-tolerant endospores) biostimulated oil bioremediation under multiple stresses by heat, oil, and heavy metals (Radwan et al., 2017).

## Materials and Methods

### Counting Heavy-Metal-Tolerant Microorganisms in Hypersaline Soil

The aim of this experiment was to study the inhibition pattern of increasing concentrations of heavy metals on the numbers of CFU of halophilic/halotolerant, hydrocarbonoclastic microorganisms counted by the dilution-plating method. A solid (1.5% agar) mineral medium with oil vapor as sole source of carbon and energy (Sorkhoh et al., 1990) was used, it had the following composition (g l^-1^): 0.85 NaNO_3_, 0.56 KH_2_PO_4_, 0.86 Na_2_HPO_4_, 0.17 K_2_SO_4_, 0.37 MgSO_4_.7H_2_O, 0.7 CaCl_2_.2H_2_O, 2.5 ml of a trace element mixture consisting of (g l^-1^): 2.32 ZnSO_4_, 1.78 MnSO_4_, 0.56 H_3_BO_3_, 1.0 CuSO_4_, 0.39 Na_2_ MoO_4_, 0.42 CoCl_2_, 0.66 KI, 1.0 EDTA, 0.4 FeSO_4_, 0.004 NiCl_2_, pH 7.0. With oil vapor as a sole carbon and energy source, this medium obviously would support hydrocarbonoclastic microorganisms selectively. The medium salinity was adjusted to 2 M NaCl, and different concentrations of individual heavy-metal salts were added. Medium portions, 25 ml, were dispensed in sterile Petri dishes. After medium solidification at room temperature, inoculum aliquots, 0.25 ml [from down series of dilutions of the hypersaline soil (about 4 M NaCl) in sterile hypersaline water, about 3 M NaCl], were spread on the solid medium surfaces. Filter papers impregnated with 2 ml crude oil as source of oil vapor were fixed in dish lids, and the dishes were sealed. The heavy-metal salts tested were HgCl_2_, CdSO_4_, PbNO_3_, CuSO_4_, and Na_2_HAsO_4_ at concentrations from 0 (controls) to 300 mg l^-1^. Three replicates for each soil dilution and heavy-metal concentration were prepared. The plates were incubated at 30°C for 12 days (d) and the CFUs were counted and the mean numbers per gram soil were calculated.

### Halophilic/Halotolerant, Hydrocarbonoclastic Microorganisms

Pure cultures of the four halophilic, hydrocarbonoclastic bacteria, *Arhodomonas aquaeolei, Marinobacter lacisalsi, Halomonas axialensis*, and *Kocuria flava*, and the two haloarchaea, *Haloferax elongans* and *Halobacterium salinarum*, that had been used in our earlier investigation (Al-Mailem et al., 2014a,b, 2017) were used in this study. These cultures had been isolated from the hypersaline coastal soil (about 4 M NaCl), about 90 km, south of Kuwait city, 10 km north of the Saudi Arabian borders, using the mineral medium with oil vapor as sole source of carbon and energy. Those isolates had been identified by comparing sequences of their 16S rRNA genes with those of type strains in the GenBank database. For this, total genomic DNA was extracted from the cells using the PrepMan Ultra Kit (Applied Biosystems, United States) and the 16S rRNA genes were amplified using the primer pairs GM5F and 907R (Santegoeds et al., 1998) for bacteria and the pairs of 0018F and 1518R for archaea (Cui et al., 2009). The PCR products were purified, sequenced, and obtained sequences were subjected to basic local alignment search tool analysis. The sequences were deposited in the GenBank database. A phylogenetic tree was constructed by neighbor joining including bootstrap analysis using PAUP^∗^ V.4 (Swofford, 1998).

### Effects of Crude Oil, Fe_2_(SO_4_)_3_, and Proline on Heavy-Metal Tolerance by Pure Halophilic/Halotolerant Isolates

Heavy-metal tolerance is measured either as “maximum tolerated concentrations” (MTCs) or as “minimum inhibitory concentrations” (MIC). The MTC measurement was chosen here because it was more related to the objectives of this study than the MIC measurement.

For this experiment, saline (2 M NaCl) nutrient agar was used, once as such, and once with oil vapor in the dish head spaces. Obviously, the mineral medium with oil vapor as a sole carbon source is not suitable because in the absence of oil vapor, the sole carbon source, no microbial activity would occur at all. Aliquots of the media were provided with individual heavy-metal salts at concentrations of 0–1600 mg l^-1^. To study the effects of Fe_2_(SO_4_)_3_ (10 mg l^-1^) and proline (200 mg l^-1^) (those concentrations were based on the results of preliminary experiments and the concentrations used by earlier researchers), they were added, separately. Common inocula (one loopful of biomass in 5 ml of sterile hypersaline water) were prepared, and the medium aliquots were inoculated by streaking one loopful of the inoculum on medium surfaces. Triplicates were prepared throughout. The cultures were incubated at 30°C for 12 d, and examined for the highest heavy-metal concentrations, above which the tested organisms failed to grow MTC.

### Effects of Fe_2_(SO_4_)_3_, Proline, and Heavy Metals on Crude-Oil Consumption by Halophilic/Halotolerant Isolates

In this experiment, the liquid mineral medium was provided with 2 M NaCl and used. Aliquots, 50 ml, dispensed in screw-caped flasks were provided with 0.3%, w/v, crude oil alone and together with the individual heavy-metal salts: HgCl_2_ (10 mg l^-1^), PbNO_3_ (10 mg l^-1^), CuSO_4_ (20 mg l^-1^), CdSO_4_ (10 mg l^-1^), and Na_2_HAsO_4_ (20 mg l^-1^). In addition, Fe_2_(SO_4_)_3_ (10 mg l^-1^) and proline (200 mg l^-1^) were amended, separately. Triplicates were prepared throughout. The media were inoculated with 1 ml portions of common inocula, sealed, and incubated at 30°C on a reciprocal shaker, 110 rpm, for 12 d. The residual oil in each flask was recovered with three 10 ml successive portions of pentane. The combined extract was raised to 35 ml using pure pentane, and 1 μl aliquots were analyzed by gas–liquid chromatography (GLC) using a Chrompack (Chrompack, Middelburg, Netherlands) CP-9000 instrument equipped with a FID, a WCOT-fused silica CP-SIL-5CB capillary column, and a temperature program, which raised the temperature from 45 to 310°C, 10°C min^-1^. The oil consumption was calculated as percent decreases of the total peak areas in the GLC profiles at the end of incubation based on the total peak areas of the abiotic controls (similarly treated, but using previously autoclaved inocula). Mean readings of the triplicates ± standard deviation values were calculated.

### Effects of Heavy Metals on Growth of Halophilic/Halotolerant Isolates at Different Salinities

In this experiment, nutrient broth was used. Medium aliquots in test tubes were provided with NaCl at concentrations from 0.0 to 4.5 M. To study the effects of heavy metals on microbial growth, the medium aliquots were provided in addition with individual heavy-metal salts ranging in concentration from 0.0 to 80 mg l^-1^ of HgCl_2_ or PbNO_3_, and from 0.0 to 160 mg l^-1^ of CdSO_4_, CuSO_4_, or Na_2_HAsO_4_. Selection of those concentrations was based on results of preliminary experiments. Triplicates were prepared throughout. The test-tube cultures were inoculated with common inocula, and incubated at 30°C for 5 d. Growth was measured as optical density values at the commonly used wavelength of 660_nm_, using a spectrophotometer (Spectronic 21D, Milton Roy, United States). The use of oil-containing medium was avoided because the water-immiscible oil would have interfered with optical density measurement.

### Effects of Salinity on Heavy-Metal Uptake by Halophilic/Halotolerant Isolates

The cells were first allowed to propagate in a heavy-metal-free medium, and were subsequently suspended in solutions of the metals whose uptake was to be measured.

Nutrient broth as a medium and the heavy-metal salts, HgCl_2_ and PbNO_3_, were used in this experiment. The oil-containing medium was not used because oil would interfere with heavy-metal uptake. Medium salinities were adjusted to 0, 1, 2, and 3 M NaCl. Heavy-metal-free medium aliquots were inoculated as described above, and incubated at 30°C for 24 h to allow for cell growth without heavy metals. Cells were harvested by centrifugation, 10,000 × *g*, for 10 min at 4°C and washed twice for a few minutes with sterile deionized water. The results of a preliminary experiment had confirmed that cells of *H. salinarum* (known to lyse in pure water) remained intact in deionized water for up to 3 h (duration of OD determination), but after 24 h complete cell lysis occurred. To recall, cell washing with water in this experiment took only minutes. The cells were subsequently suspended in 50 ml portions of deionized water containing 0, 1, 2, or 3 M NaCl, 0.2%, w/v, glucose and either HgCl_2_ (10 mg l^-1^) or PbNO_3_ (10 mg l^-1^). The suspensions were incubated for 40 min at 30°C. Longer incubation could have led to cell lysis after 3 h (at 0 M NaCl) as the preliminary experiments revealed. At time 0 and in 5 min intervals, 5 ml samples were harvested and centrifuged (10,000 × *g*) for 2 min at 4°C. Cells were digested with conc. HNO_3_ and HCl, 2:1 on a hot plate. The digestion solutions were filtered, diluted with 1% HNO_3_, and the heavy-metal-salt concentrations therein were determined by the USEPA method 6010B using Inductively Coupled Plasma Optical Emission Spectrometry (ICP-OES; PerkinElmer Optima 7300DV, United States) and Certified Reference Materials (multi-elements) (Oyetibo et al., 2010). As blank, the uninoculated liquid medium containing all the reagents was used. Reportedly, this method produces an average heavy-metal recovery of 100% with only 0.92% relative error (Han et al., 2006). The differences from the start concentrations were considered as the heavy-metal uptake values.

### Statistical Analysis

As already mentioned, three parallel replicates for each analysis were prepared throughout, and the mean values ± standard deviation values were calculated using Microsoft Excel 2003. Statistical Package for Social Sciences, version 12 was used to assess the degree of significance, where the analysis of variance (ANOVA) was used to differentiate between the means of the tested parameters.

## Results and Discussion

### Numbers of Cultivable Heavy-Metal-Resistant CFU in Hypersaline Soil

To recall, the culture-dependent analysis captures the cultivable, hydrocarbonoclastic microorganisms only. Heavy metals are grouped into toxic and less toxic ones (Massadeh et al., 2005; Salgaonkar et al., 2013). This study focused on the first group that comprises mercury, cadmium, lead, and arsenic. For comparison, two representatives of the much less toxic heavy metals, copper and zinc, were used.

**FIGURE 1:**
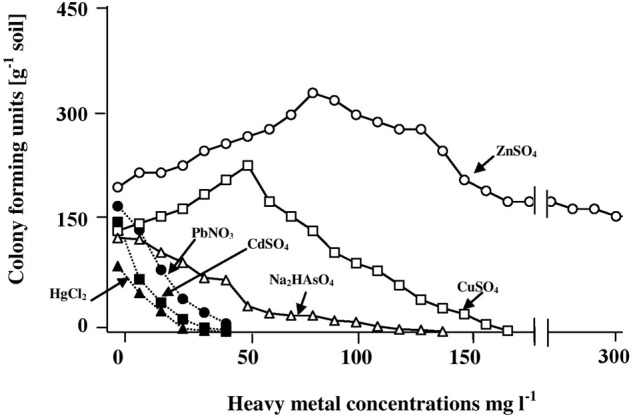
Numbers of CFU of hydrocarbonoclastic microorganisms in hypersaline soil as determined by the dilution-plating method on heavy-metal containing mineral medium with oil vapor as a sole source of carbon and energy. The medium salinity was adjusted to 2 M NaCl (section “Materials and Methods”). The CFU numbers are obviously those of cultivable microorganism only.

The results in **Figure 1** show that the CFU numbers of hydrocarbonoclastic microorganisms (capable of growth on the mineral medium with oil vapor as a sole source of carbon and energy) as counted on the heavy-metal-free medium (control) ranged between about 170 ± 7 and 200 ± 11 CFU g^-1^. Addition of salts of mercury, cadmium, lead, and arsenic led to the lowest numbers of CFU counted. Increasing concentrations of those salts led to immediate decreases of the CFU numbers, until no more colonies appeared at heavy-metal-salt concentrations around 40 mg l^-1^. Basically, the same result was obtained with Na_2_HAsO_4_, although the maximum concentration of this heavy-metal salt above which no CFU appeared was >100 mg l^-1^. The results with CuSO_4_ and ZnSO_4_ were quite different. Within the concentration ranges of 0–50 and 80 mg l^-1^ of those salts, respectively, concentration increases were associated with immediate increases in the CFU numbers. Maximum numbers were counted in the presence of 50 mg l^-1^ CuSO_4_ (a concentration at which Hg^2+^, Cd^2+^, and Pb^+^ prevented CFU growth) and about 80 mg l^-1^ ZnSO_4_. Those enhancing effects are probably related to the roles of Cu^2+^ and Zn^2+^ in certain enzymatic activities (Salgaonkar et al., 2013). Furthermore, Cu^2+^ and Zn^2+^ with their low toxicity may be involved in cytoplasmic osmolarity regulation under high salinity, just like the conventional cations, Na^+^ and K^+^. Copper was more potent in its inhibitory effect than zinc, the maximum CuSO_4_ concentration above which no CFU appeared was 140 mg l^-1^, while as high as 300 mg l^-1^ ZnSO_4_ did not result in reducing the CFU numbers below those counted in the complete absence of this salt.

### Pure Isolates of Hydrocarbonoclastic Microorganisms

**FIGURE 2:**
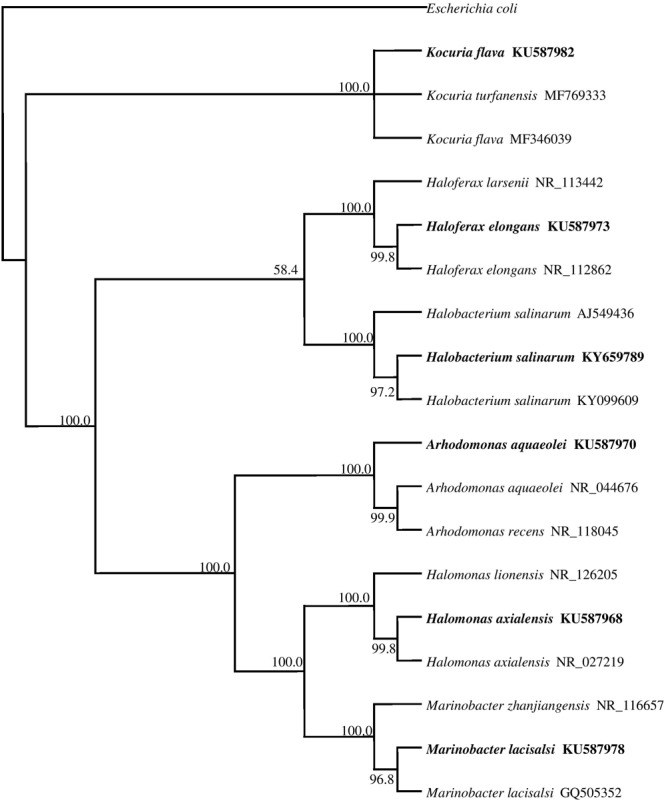
16S rRNA gene phylogeny of hydrocarbonoclastic bacterial and archaeal isolates used in this study. Values used in each node of the tree are bootstrap values; 2000 bootstrap replicates were performed. The tree includes the accession numbers of the isolates.

The phylogenetic tree in **Figure 2** shows the phylogenetic affiliations among the pure isolates studies and includes their accession numbers in the GenBank database.

### Heavy-Metal Tolerance by Halophilic/Halotolerant Isolates on Oil-Free and Oil-Containing Media

**FIGURE 3:**
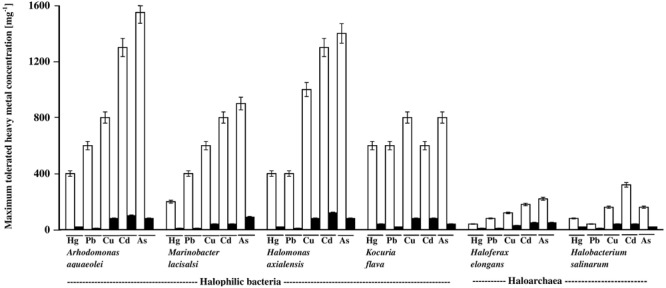
Heavy-metal tolerance by halophilic/halotolerant bacterial and archaeal cultures in the presence (black columns) and absence (white columns) of crude oil. Hg, HgCl_2_; Pb, PbNO_3_; Cu, CuSO_4_; Cd, CdSO_4_; As, Na_2_HAsO_4_. Each reading was the mean of three parallel replicates, whose maximum tolerated values were identical, i.e., no deviation. The medium salinity was adjusted to 2 M NaCl (section “Materials and Methods”).

The histograms in **Figure 3** illustrate the dramatic inhibitory effects of crude oil on the heavy-metal tolerance by the four hydrocarbonoclastic bacterial and the two hydrocarbonoclastic haloarchaeal species from the hypersaline soil. Interestingly, the two haloarchaeal species seemed to be more sensitive to the tested heavy metals (showing weaker growth) than the four halophilic bacterial species, particularly on the crude-oil-free nutrient agar. Earlier researchers noted that numerous halophilic bacteria but only a few haloarchaea possess heavy-metal resistance (for review, see Voica et al., 2016). The highest tolerance measured in our study was that of *A. aquaeolei* which tolerated up to 1600 mg l^-1^ Na_2_HAsO_4_, and the lowest was that of *M. lacisalci* which tolerated up to only 200 mg l^-1^ HgCl_2_. High tolerance values were also measured for *H. axialensis*, which tolerated up to 1200 mg l^-1^ of CdSO_4_, CuSO_4_, and Na_2_HAsO_4_. *K. flava* tolerated up to 800 mg l^-1^ of CuSO_4_ and Na_2_HAsO_4_ and up to 600 mg l^-1^ of HgCl_2_, CdSO_4_, and PbNO_3_. In contrast, the highest tolerance of the haloarchaeon, *H. elongans* was at 200 mg l^-1^ Na_2_HAsO_4_, and that of *H. salinarum* was at 320 mg l^-1^ CdSO_4_. The heavy-metal-tolerance values on the oil-containing medium were lower than on the oil-free medium. A simple calculation showed that the highest concentrations of heavy metals tolerated by bacteria in the presence of oil were between only about 3 and 10% (in one case only reaching 13%) of the corresponding values in the absence of oil (on nutrient agar). Corresponding tolerance values for the archaeal species were considerably higher, between 20 and 25% (with 13% in three cases only).

There are no relevant result in the available literature to compare them with the above results. However, the weaker heavy-metal tolerance in the presence of oil than in its absence may be due to additional stress by aromatic hydrocarbons in the oil used (Shukla and Singh, 2012).

### Effects of Fe_2_(SO_4_)_3_ and Proline on the “Maximum Heavy-Metal Concentrations” Tolerated by Halophilic/Halotolerant Isolates

**FIGURE 4:**
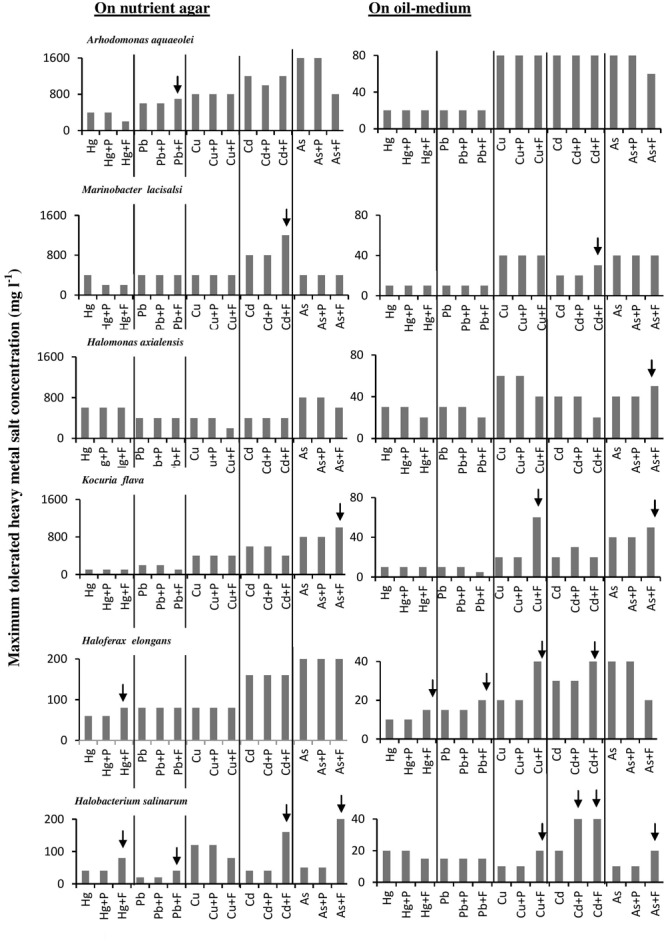
Effects of Fe^3+^ and proline on maximum-heavy-metal concentrations tolerated by halophilic/ halotolerant microorganisms. Arrows highlight positive effects of added Fe^3+^ and proline. Hg, HgCl_2_; Pb, PbNO_3_; Cu, CuSO_4_; Cd, CdSO_4_; As, Na_2_HAsO_4_. Each reading was the mean of three parallel replicates, whose maximum tolerated values were identical, i.e., there were no deviations. The medium salinity was adjusted to 2 M NaCl (section “Materials and Methods”).

The histograms in **Figure 4** confirm that bacterial cultures commonly tolerated higher concentrations of heavy metals than archaeal cultures. The data also show that the tested microorganisms tolerated higher heavy-metal concentrations on the oil-free than in the oil-containing media. In many cases (highlighted by arrows in **Figure 4**), the addition of Fe^3+^ and, albeit to a less extent, proline enhanced this tolerance. The highest tolerated heavy-metal concentrations were double to triple as high as the concentrations tolerated in the absence of Fe_2_(SO_4_)_3_ and proline. This result was more frequent among the tested haloarchaea than among the halophilic bacteria. In several other cases, Fe_2_(SO_4_)_3_ and proline did not affect the heavy-metal tolerance at all. There are no relevant results in the available literature to compare them with those results. However, Fe^3+^ and proline might have contributed to osmoregulation of the cytoplasm leading to less salt stress and thus to enhanced heavy-metal tolerance. Why this did not happen in several other cases as **Figure 4** shows, this could be due to the fact that concerned microorganisms accumulate other compatible solutes for osmoregulation.

### Effects of Fe_2_(SO_4_)_3_ and Proline on Heavy-Metal-Mediated Inhibition of Crude-Oil Consumption by Halophilic/Halotolerant Isolates

**FIGURE 5:**
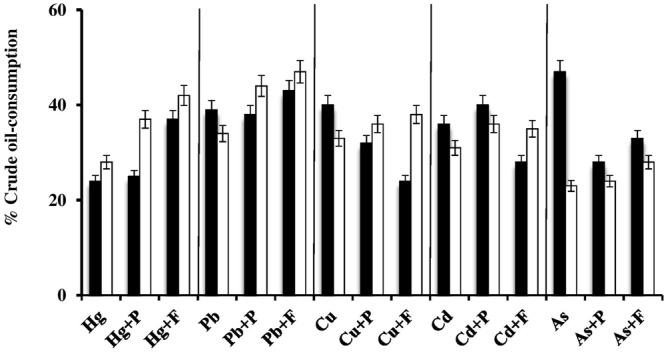
Effects of Fe^3+^ and proline on heavy-metal-mediated inhibition of crude oil consumption by the halophilic/halotolerant bacterium, *Kocuria flava* (black columns) and the haloarchaeon, *Halobacterium salinarum* (white columns). Hg, HgCl_2_; Pb, PbNO_3_; Cu, CuSO_4_; Cd, CdSO_4_; As, Na_2_HAsO_4_; P, proline; F, Fe_2_(SO_4_)_3_. The medium salinity was adjusted to 2 M NaCl (section “Materials and Methods”).

In view of the extensive setup, this experiment was done on one bacterial and one haloarchaeal species only. The histograms in **Figure 5** show that, in most of the cases, Fe^3+^ and proline significantly (*P* < 0.05) enhanced the oil consumption by the studied microorganisms in the presence of heavy metals. In many cases, the Fe^3+^-amendments led to consumption values much higher even than those in the absence of heavy metals. Still in some other cases, these amendments had even negative effects on oil consumption. Again the enhancement of oil consumption could have been due to the osmoregulatory effects of Fe^3+^ and proline, thus reducing the salt stress and supporting the heavy-metal tolerance of the respective oil-utilizing microorganisms. Furthermore, it has been reported that Fe^3+^ may serve as an electron acceptor during anaerobic respiration of bacteria and archaea (Lovley et al., 1991).

**FIGURE 6:**
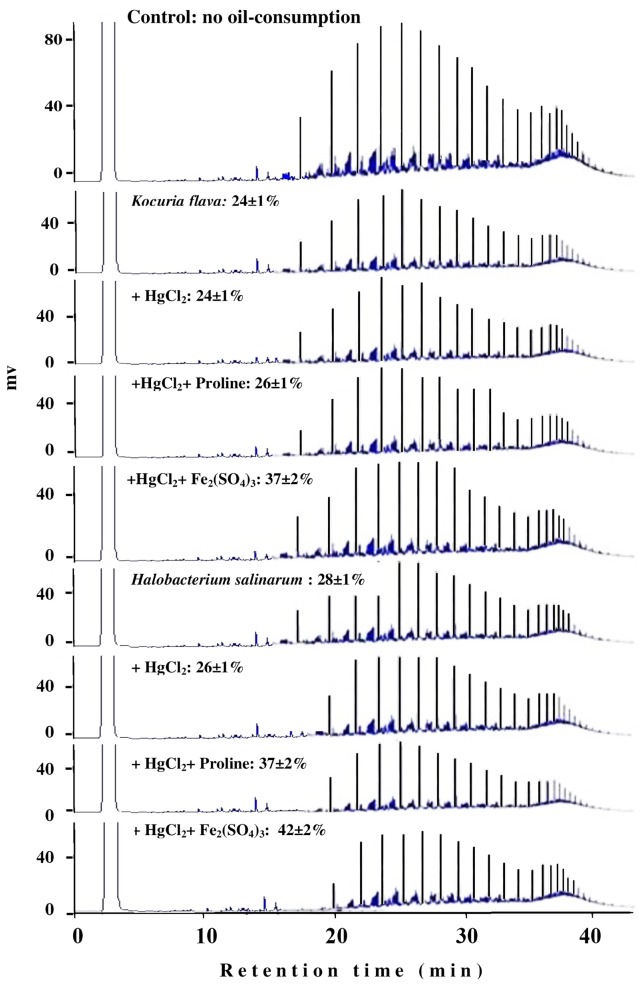
Typical GLC profiles of residual hydrocarbons in microbial cultures treated with Fe_2_(SO_4_)_3_ and proline. Percent values on the individual profiles are those of oil-consumption ± standard deviation. Note that small peaks mean more consumption, and that amendment with Fe_2_(SO_4_)_3_ and proline significantly enhanced the oil-consumption values by both organisms compared with the values in the presence of the heavy metals alone.

The typical GLC profiles in **Figure 6** illustrate this novel result using the most toxic heavy-metal Hg^2+^. Irrespective of the actual mechanism(s) by which Fe^3+^ and proline may enhance the oil consumption by some microorganism, this result could obviously be of practical value in designing bioremediation biotechnologies for oil-contaminated, hypersaline environments.

### Effects of Heavy Metals on the Growth Magnitudes of Halophilic/Halotolerant Microorganisms Under Salt Stress

**FIGURE 7:**
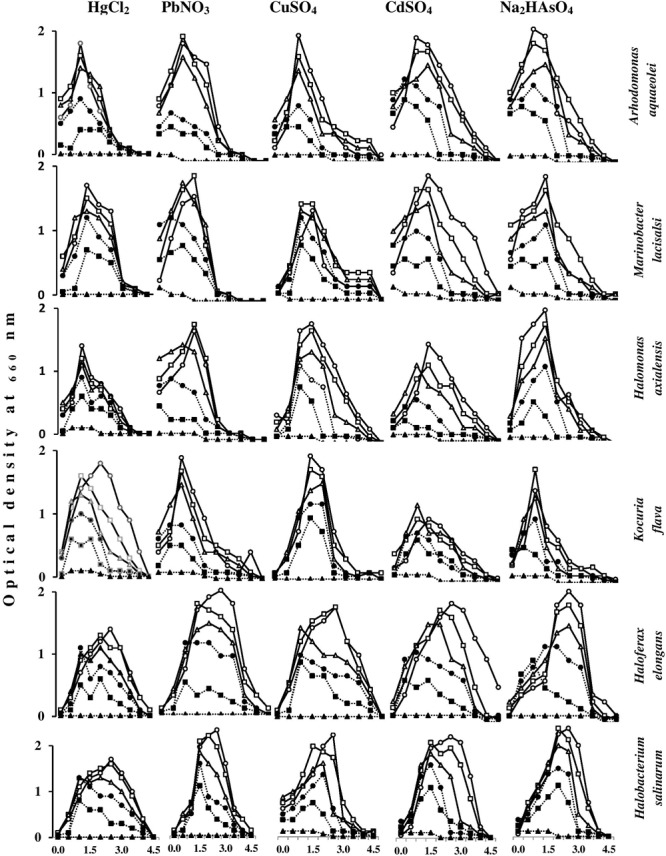
Effects of heavy-metal concentrations on the growth magnitudes of halophilic/halotolerant isolates at different salinities. HgCl_2_ and PbNO_3_ concentration (mg l^-1^): -o-, 0; -□-, 5; -Δ-, 10; -●-, 20; -■-, 40; -▲-, 80. CuSO_4_, CdSO_4_, Na_2_HAsO_4_ concentration (mg l^-1^): -o-, 0; -□-, 10; -Δ-, 20; -●-, 40; -■-, 80; -▲-, 160.

**Figure 7** shows the effects of heavy metals on the growth magnitudes of tested isolates in nutrient broth containing various NaCl concentrations. The heavy metals did not only affect the growth magnitudes but also resulted in changing the optimum NaCl concentrations for growth of some isolates. As should be expected, the growth of the tested species was frequently best in the absence of heavy metals, and decreased with increasing heavy-metal concentrations. Exceptions, which are obvious in this figure, are quite interesting. Thus, with some organisms, HgCl_2_ and PbNO_3_ concentrations of 5 and 10 mg l^-1^ at certain salinities resulted in growth values similar to or higher than the values in the absence of those heavy metals. This was also true for CdSO_4_, CuSO_4_, and Na_2_HAsO_4_ at concentrations of 10 and 20 mg l^-1^. Higher heavy-metal-salt concentrations of up to 40 mg l^-1^ HgCl_2_ and up to 80 mg l^-1^ of all other heavy-metal salts did not arrest the growth of the tested organisms at most of the salinities. These results may imply that the “toxic” heavy-metal cations at low concentrations may be beneficial, probably by affecting cytoplasmic osmolality under environmental salt stress, like conventional cations do (K^+^, Mg^2+^, Ca^2+^, and Fe^3+^) (Al-Mailem et al., 2017). Furthermore, toxic heavy metals at trace concentrations were reported to serve as essential micronutrients in metabolic reactions and enzyme stabilization (Bruins et al., 2000).

### Heavy-Metal Uptake by Halophilic/Halotolerant Isolates at Various Salinities

It is important to recall that this experiment extended for only 40 min, and that it took haloarchaeal cells at least 3 h to start lysis in the absence of NaCl (see above). The results proved, however, that this short period was adequate to fulfill the objectives targeted.

**FIGURE 8:**
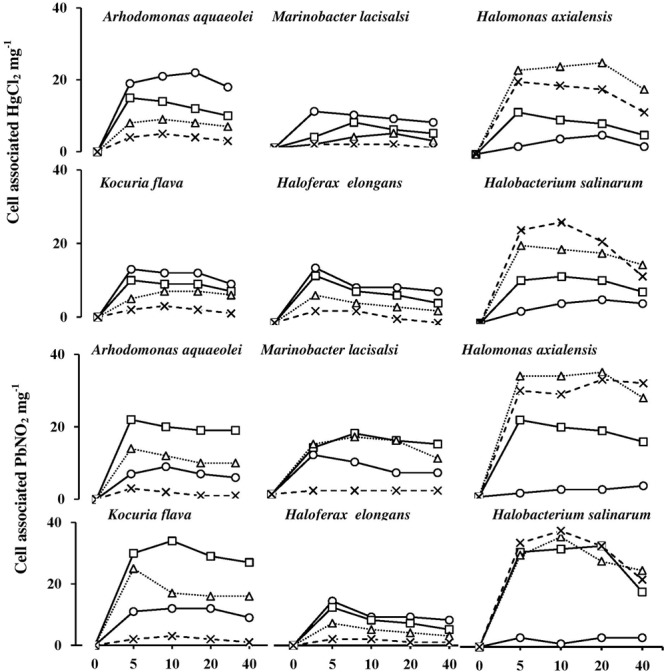
Heavy-metal uptake by halophilic microorganisms at various salinities. NaCl concentrations (M): circles, 0 M; squares, 1 M; triangles, 2 M; cross, 3 M.

**Figure 8** shows the uptake rates of HgCl_2_ and PbNO_3_ by the six tested isolates. The uptake of both salts was almost completed during the first 5 min of incubation. Their cell-associated concentrations remained nearly constant or exhibited some decreases with time. The smallest proportions of HgCl_2_ and PbNO_3_ were taken up by the bacterium, *H. axialensis* and the haloarchaeon, *H. salinarum* in the absence of NaCl, whereas the highest uptake values by both microorganisms were in the hypersaline range (2 and 3 M NaCl). Conversely, the greatest amounts of HgCl_2_ were taken up in the absence of NaCl by *A. aqueoleoi, M. lacisalsi, K. flava*, and *H. elongans*; the latter species also took up the greatest amount of PbNO_3_ in the absence of NaCl. In the remaining cases, the highest concentrations of the two studied heavy-metal salts were taken up at 1 and 2 M NaCl, but 3 M NaCl inhibited the heavy-metal uptake. The fact that there was a direct relationship between salinity and heavy-metal uptake consolidates the assumption that heavy-metal cations may play some role in affecting cytoplasmic osmolality under salt stress. It may be argued that the heavy metals were just adsorbed on the cell envelops as it happens in many microorganisms as a part of the heavy-metal tolerance strategy (Nies, 1999; Deb et al., 2013). However, the tolerance of Hg^2+^, one of the two studied metals, is known to follow a completely different strategy which involves the reduction of the toxic Hg^2+^ to the less toxic volatile Hg^o^ form (Nies, 1999; Wiatrowski et al., 2006; De et al., 2008). The patterns of HgCl_2_ uptake in **Figure 8**, however, are more similar to the active-transport pattern than to the pattern of metal loss by volatilization.

## Conclusion

The results of this study provide novel information complementary to our recent findings on biostimulation for oil bioremediation in salt-stressed environments. Although hydrocarbonoclastic bacteria and archaea indigenous to hypersaline soil occur permanently under multiple stresses, they still maintain the potential for active growth and biodegradation of spilled oil. In batch cultures, those organisms proved capable of growth and crude-oil removal in the presence of certain concentrations of heavy metals and high NaCl concentrations. For some isolates, those activities were significantly biostimulated by amended substances. Thus, Fe^3+^ and proline enhanced the tolerance of some organisms to toxic heavy metals, thus raising their potential for spilled-oil bioremediation under the prevailing multiple stresses. This study offers experimental evidence for quick uptake of heavy metals by tested microorganisms under salt stress. Although toxic, some metals enhanced salt tolerance and biodegradation of spilled oil in hypersaline environments.

## Authors Contributions

DA-M, ME, and SR drafted the manuscript and performed the microbiological studies and genome sequencing analysis. DA-M and SR participated in the design of the study. All authors read and approved the final manuscript.

## Conflict of Interest Statement

The authors declare that the research was conducted in the absence of any commercial or financial relationships that could be construed as a potential conflict of interest.
